# Implementing collaborative care for depression treatment in primary care: A cluster randomized evaluation of a quality improvement practice redesign

**DOI:** 10.1186/1748-5908-6-121

**Published:** 2011-10-27

**Authors:** Edmund F Chaney, Lisa V Rubenstein, Chuan-Fen Liu, Elizabeth M Yano, Cory Bolkan, Martin Lee, Barbara Simon, Andy Lanto, Bradford Felker, Jane Uman

**Affiliations:** 1 Department of Psychiatry and Behavioral Sciences, School of Medicine, University of Washington, Seattle Washington, USA; 2 VA Center for the Study of Healthcare Provider Behavior, VA Greater Los Angeles Healthcare System, Los Angeles, California, USA; 3 RAND Health Program, Santa Monica, California, USA; 4 David Geffen School of Medicine and School of Public Health, University of California Los Angeles, Los Angeles, California, USA; 5 HSR&D Northwest Center of Excellence for Outcomes Research in Older Adults, VA Puget Sound Health Care System, Seattle, Washington, USA; 6 Department of Human Development, Washington State University, Vancouver, Washington, USA; 7 VA Puget Sound Health Care System, Seattle, Washington, USA

## Abstract

**Background:**

Meta-analyses show collaborative care models (CCMs) with nurse care management are effective for improving primary care for depression. This study aimed to develop CCM approaches that could be sustained and spread within Veterans Affairs (VA). Evidence-based quality improvement (EBQI) uses QI approaches within a research/clinical partnership to redesign care. The study used EBQI methods for CCM redesign, tested the effectiveness of the locally adapted model as implemented, and assessed the contextual factors shaping intervention effectiveness.

**Methods:**

The study intervention is EBQI as applied to CCM implementation. The study uses a cluster randomized design as a formative evaluation tool to test and improve the effectiveness of the redesign process, with seven intervention and three non-intervention VA primary care practices in five different states. The primary study outcome is patient antidepressant use. The context evaluation is descriptive and uses subgroup analysis. The primary context evaluation measure is naturalistic primary care clinician (PCC) predilection to adopt CCM.

For the randomized evaluation, trained telephone research interviewers enrolled consecutive primary care patients with major depression in the evaluation, referred enrolled patients in intervention practices to the implemented CCM, and re-surveyed at seven months.

**Results:**

Interviewers enrolled 288 CCM site and 258 non-CCM site patients. Enrolled intervention site patients were more likely to receive appropriate antidepressant care (66% versus 43%, p = 0.01), but showed no significant difference in symptom improvement compared to usual care. In terms of context, only 40% of enrolled patients received complete care management per protocol. PCC predilection to adopt CCM had substantial effects on patient participation, with patients belonging to early adopter clinicians completing adequate care manager follow-up significantly more often than patients of clinicians with low predilection to adopt CCM (74% versus 48%%, p = 0.003).

**Conclusions:**

Depression CCM designed and implemented by primary care practices using EBQI improved antidepressant initiation. Combining QI methods with a randomized evaluation proved challenging, but enabled new insights into the process of translating research-based CCM into practice. Future research on the effects of PCC attitudes and skills on CCM results, as well as on enhancing the link between improved antidepressant use and symptom outcomes, is needed.

**Trial Registration:**

ClinicalTrials.gov: NCT00105820

## Background

Despite efficacious therapies, depression remains a leading cause of disability [1-3]. Most depression is detected in primary care, yet rates of appropriate treatment for detected patients remain low. There is ample randomized trial evidence that collaborative care management (CCM) for depression is an effective [4-6] and cost-effective [7] approach to improving treatment and outcomes for these patients. In CCM, a care manager supports primary care clinicians (PCCs) in assessing and treating depression symptoms, often with active involvement of collaborating mental health specialists. Care managers typically carry out a comprehensive initial assessment followed by a series of subsequent contacts focusing on treatment adherence and patient education and/or activation. Use of CCM, however, is not yet widespread in routine primary care settings. This study aimed to use a cluster-randomized design to formatively evaluate the success of evidence-based quality improvement (EBQI) methods in implementing effective CCM as part of routine Veteran Affairs (VA) care. Problems detected through our rigorous evaluation could then be used to support higher quality model development for sustaining and spreading CCM in VA primary care practices nationally. The study's major goals were thus: to learn about the process of implementing research in practice, including effects of context; to test the effectiveness of EBQI for adapting research-based CCM while maintaining its effectiveness; and to provide information for improving the implemented model.

Implementation of CCM as part of routine primary care requires system redesign. EBQI is a redesign method that supports clinical managers in making use of prior evidence on effective care models while taking account of local context [8-10]. For this study, VA regional leaders and participating local sites adapted CCM to VA system and site conditions using EBQI [11]. We term the locally adapted CCM model EBQI-CCM. The study used a cluster-randomized design to evaluate seven EBQI-CCM primary care practices versus three equivalent practices without EBQI-CCM.

Theory suggests that durable organizational change of the kind required by CCM is most likely when stakeholders are involved in design and implementation [12,13]. Yet classical continuous quality improvement (CQI) for depression, which maximizes participation, does not improve outcomes [14,15]. EBQI, a more structured form of CQI that engages leaders and QI teams in setting depression care priorities and understanding the evidence base and focuses teams on adapting existing CCM evidence and tools, has been more successful [8,16-18]. This study built on previous EBQI studies by adding external technical expert support from the research team to leverage the efforts of QI teams [19].

CCM can be considered a practice innovation. Research shows that early adopters of innovations may be different from those who lag in using the innovation [20]. We hypothesized that CCM, which depends on PCC participation, might yield different outcomes for patients of early adopter clinicians compared to patients of clinicians who demonstrated less use of the model. We found no prior CCM studies on this topic. Because this study tested a CCM model that was implemented as routine care prior to and during the randomized trial reported here, we were able to classify clinicians in terms of predilection to adopt CCM based on their observed model participation outside of the randomized trial. We then assessed CCM outcomes for our enrolled patient sample as a function of their PCC's predilection to adopt the model.

In this paper, we evaluate implementation by asking the intent to treat question: did depressed EBQI-CCM practice patients enrolled in the randomized evaluation and referred to CCM have better care than depressed patients at practices not implementing CCM? We also asked the contextual subgroup question: do EBQI-CCM site patients of early adopter clinicians experience different CCM participation outcomes than those of clinicians with a low predilection to adopt CCM? Because our purpose was to study and formatively evaluate the implementation of a well-researched technology [5], our grant proposal powered the study on a process of care change (antidepressant use). We also assessed pre-post depression symptom outcome data on all patients referred to care management as part of EBQI. This data is documented elsewhere [11], and is used in this paper to gain insight into differences between naturalistically-referred patients (representing true routine care use of CCM in the sites outside of research) and study enrolled patients.

## Methods

This study evaluated EBQI-CCM implementation through a cluster-randomized trial. The EBQI process occurred in seven randomly allocated group practices in three VA multi-state administrative regions; three additional practices (one in each region) were simultaneously selected to serve as comparison sites in the subsequent cluster randomized evaluation reported here. Primary care providers began referring their patients to EBQI-CCM through VA's usual computer-based consult system a year prior to any patient enrollment in the randomized evaluation as part of the ongoing TIDES (Translating Initiatives in Depression into Effective Solutions) QI program [19]. Additional information on the EBQI process and QI outcomes is available [11].

### Setting

Researchers formed partnerships with three volunteer Veterans Health Administration regions between 2001 and 2002 to foster a CCM implementation QI project (TIDES). Participating regions spanned 19 states in the Midwest and South. Regional directors agreed to engage their mental health, primary care, QI, and nursing leadership in EBQI teams for improving depression care, and to provide release time to enable team members to participate. Each region agreed to hire a care manager for depression. Researchers provided dollars totaling the equivalent of one halftime nurse care manager for 21 months to each region.

Prior to initiation of EBQI, each regional administrator agreed to identify three primary care practices of similar size, availability of mental health personnel, and patient population profiles for participation in the study. Study practices mirrored staffing characteristics of small to medium-sized non-academic practices nationally in VA. As described elsewhere, however, baseline levels of participation in care for depression in primary care varied [21].

### Randomization

In 2002, the study statistician randomly assigned one practice per region as control practices, and the remaining two practices per region to EBQI-CCM. One of the six EBQI-CCM sites selected by regional administrators was a single administrative entity but composed of two geographically separated practices with different staffing. We therefore analyzed it as two separate practices for a total of seven EBQI-CCM sites.

### Human Subjects Protection

All study procedures for the QI process and for the randomized evaluation were approved by Institutional Review Boards (IRBs) at each participating site and at each site housing investigators (a total of eight IRBs).

### EBQI Intervention

We described the steps, or phases, in the TIDES EBQI process and their cost in prior publications [19]. These include: preparation (leadership engagement); design (developing a basic design at the regional level and engaging local practices); and implementation (Plan-Do-Study-Act or PDSA cycles to refine CCM until it becomes stable as part of routine care). The randomized trial reported here began during the early implementation phase of TIDES.

During preparation (approximately 2001 and 2002), each region learned about the project and identified its regional leadership team. During the design phase, the regional leadership team and representatives from some local sites carried out a modified Delphi panel [8,22] to set CCM design features. For example, two out of three regions chose primarily telephone-based rather than in-person care management [23], reflecting concern for providing mental health access to rural veterans. The remaining region switched to this approach after initial PDSA cycles.

The implementation phase began with enrollment of the first patient in a PDSA cycle. After the depression care manager (DCM) was designated or hired, she and a single PCC initially worked together to plan and implement rapid enlarging PDSA cycles that aimed to test the referral process, safety, process of depression care, and outcomes. Cycles began with one patient and one clinician in each participating practice. After several cycles (*e.g*., 10 to 15 patients) care managers began engaging additional clinicians and patients through academic detailing and local seminars [24,25]. A total of 485 patients had entered CCM by June 2003 prior to the start of the randomized trial. Thus, in all practices, the EBQI-designed CCM model was part of routine care before recruitment for the randomized evaluation began [19]. When randomized trial enrollment began, care manager workloads were in equilibrium, with similar numbers of patients entering and exiting CCM. During and after the trial improvement work continued, with for instance a focus on care manager electronic decision support, training, and methods for engaging primary care providers, but at a gradual pace.

PDSA cycles require aims, measures, and feedback. Initial aims focused on successful development of program components. For example, for decision support, PDSA cycles assessed questions such as: Is the DCM's initial assessment capturing information necessary for treatment planning? Are DCMs activating patients [26]? How usable is EBQI-CCM information technology for consultation, assessment, and follow-up [27-29]? For patient safety, cycles asked: Is there a working suicide risk management protocol in place [30]? Later cycles focused on how to best publicize the intervention and educate staff and how to best manage more complicated patients through collaboration with mental health specialists [31,32]. Throughout all cycles, DCMs monitored patient process of care and outcomes.

In terms of measures, we rigorously trained DCMs to administer instruments (*e.g*., the PHQ-9) and keep registries of patient process and outcomes. Registry data provided measures for overarching quarterly PDSA cycles focused on patient enrollment (*e.g*., patients referred versus enrolled), patient process of care (*e.g*., treatments, location of care in primary care or mental health specialty), and patient symptom outcomes.

PDSA cycles involved feedback to participants. Interdisciplinary workgroups were the major forum for sharing and discussing PDSA results [31]. In the care management and patient self-management support workgroup, care managers met weekly for an hour by phone. Lead mental health specialists and lead PCCs met monthly in the collaborative care workgroup, while regional leaders (administrative, mental health, and primary care) met quarterly in the senior leader workgroup. Study team members assisted in administratively supporting the workgroups, reviewing cycle results, and supporting improvement design.

The study team fed back results for quarterly site-level PDSAs on patient process and outcomes to care managers, primary care, mental health, and administrative leaders at practice, medical center, and Veteran's Integrated Service Network (VISN) levels. Quarterly reports were formatted like laboratory tests, with a graph of patient outcome results; an example report is included in a previous publication [11].

### Randomized evaluation sample

Researchers created a database of potential patient evaluation participants from CCM and non-CCM practices using VA electronic medical records. Inclusion criteria were having at least one primary care appointment in the preceding 12 months in a participating practice, and having one pending appointment scheduled within the three months post-selection (n = 28, 474). Exclusion criteria were having conditions that required urgent care (acute suicidality, psychosis), inability to communicate over the telephone, or prior naturalistic referral by the patient's PCC to the DCM.

### Data collection

Trained interviewers from California Survey Research Services Inc. (CSRS) screened eligible patients for depression or dysthymia symptoms between June 2003 and June 2004 using the first two questions of the PHQ-9 [33] by telephone interview. Interviewers administered the balance of the PHQ-9 to screen positive patients, and enrolled those with probable major depression based on a PHQ-9 score of 10 or above. Interviewers referred eligible and consenting evaluation patients to the appropriate DCM for treatment. Evaluation patients were re-surveyed by CSRS at seven months post-enrollment, between March 2004 and February 2005. Health Insurance Portability and Accountability Act (HIPAA) rules introduced during the study required changes in the consent process for administrative data analysis: we re-consented willing patients at the seven-month survey.

### Depression Care Management Protocol

Both patients naturalistically referred to CCM prior to and during the study and patients referred to CCM as part of the randomized evaluation were followed by DCMs according to the TIDES care manager protocol. The protocol, developed by participating experts and sites, defined patients who had probable major depression (defined as an initial PHQ-9 greater than or equal to 10) as eligible for six months of DCM panel management. Patients with subthreshold depression (an initial PHQ-9 between five and nine) who also had a) a prior history of depression, or b) dysthymia were also eligible. Patients who entered into mental health specialty care could be discharged from the panel after the initial assessment and any needed follow-up to ensure successful referral. All others were to receive at least four care manager follow-up calls that included patient self-management support and PHQ-9 measurement. All panel-eligible patients were to be called and re-assessed by the DCM at six months. The protocol specified that any patient not eligible for or who discontinued care management be referred back to the primary care provider with individualized resource and management suggestions.

### Power Calculations

Design power calculations indicated that to detect a 50% improvement in anti-depressant prescribing assuming an intra-class correlation coefficient (ICC) of 0.01, and nine sites, with 46 patients per site, the study would have about 81% power using a two-sided 5% significance level. To allow for 20% attrition, 56 patients needed to be enrolled from each site. During data collection, new studies indicated the assumed ICC might have been too small, so within budgetary limitations, the sampling from CCM practices was increased to 386 and non-CCM practices to 375. Post-power calculations showed that the actual ICC was 0.028 and the within-group standard deviation 6.25, suggesting there was adequate power (0.96) to detect a 20% difference in anti-depressant prescribing between the two study arms, but not enough power (0.45) to detect a 10% difference. Power calculations for detecting a difference in depression symptom improvement across the two study arms show a posteriori power to detect a 20% difference between the two study arms of between 0.21 and 0.29.

### Survey and administrative data measures

Our primary study outcome measure, and the basis for our power calculations, was receipt of appropriate treatment, a process of care goal that requires less power than that required to demonstrate symptom outcome improvement. Previous studies [5] had demonstrated process/outcome links [34] for collaborative care with appropriate antidepressant use and depression symptom and quality of life improvements. For this QI study, we therefore aimed at a sample suitable for showing process change. We evaluated depression symptoms using the PHQ-9 [33] and quality of life changes using SF36V2 [35]. We also assessed physical and emotional healthcare satisfaction [36]. For evaluation patients whose consent allowed us access to their electronic medical records, we constructed adherence measures based on VA administrative databases. For these patients, we measured antidepressant availability from the VA Pharmacy Benefits Management and mental health specialty visits from VA's Outpatient Care file. We used two measures: whether a patient had any antidepressant fill at appropriate dosage in the seven-month time period, and the medication possession ratio (MPR) [37]. The MPR is defined as the proportion of days that patients had antidepressants in hand during the seven-month time period. We defined receipt of appropriate treatment as either having an antidepressant fill at or above minimum therapeutic dosage and achieving an MPR of 0.8 or having four or more mental health specialty visits [38].

Our covariate measures included baseline measures of depression symptoms, functioning, satisfaction, and adherence as described above, as well as other variables hypothesized to affect outcomes. These included dysthymia [39], history of medications for bipolar disorder, anxiety [38], post-traumatic stress disorder (PTSD) [40], alcohol use [41], and medical co-morbidity [42]. Alternatively, we used a slightly modified version of the Depression Prognostic Index (DPI) [43].

### Evaluation of impacts of clinician early adopter status as a contextual factor

#### Data collection

We trained DCMs to collect registry information on all patients referred to them and used it to prepare quarterly reports to regional and site managers. DCMs entered data, including PHQ-9 results, on each patient referred to them (including those referred by research interviewers) into a Microsoft Excel-based depression registry. Care managers recorded, de-identified, and then transmitted registry data. DCMs transmitted data on 974 patients between the date of the first PDSA cycle and the end date of the randomized evaluation. Recorded data included whether the patient had been naturalistically referred or referred as part of the randomized evaluation, and indicated the patient's PCC, but no patient personal health information identifiers such as age. The project statistician replaced clinician names with assigned study codes that linked clinicians to practice site only, without additional information.

#### Care manager registry-based measures

We used the number of naturalistic referrals (*i.e*., those carried out as part of routine care outside of the randomized evaluation) recorded in the registry for each PCC to characterize PCC adopter status [20]. We categorized these clinicians into four groups, based on number of referrals to CCM. We designated clinicians who never chose to refer outside of the randomized evaluation as having a low predilection to adopt the model (no referrals). We classified clinicians with one to four referrals as CCM slow adopters, and over five referrals as CCM early adopters. We classified clinicians who had chosen to make more than ten referrals as habitual CCM users. These cut-points reflect the distribution of the variable as well as the clinical judgment that five referrals provide substantial experience and ten referrals indicates that referral has become the PCC's routine.

We used the registry results to identify both randomized evaluation and naturalistically referred patients eligible for panel management per the TIDES care manager protocol. We also defined adequate care manager follow-up of panel-eligible patients based on the TIDES DCM protocol for follow-up, such that, for example, a patient who required four DCM follow-up visits was judged as having adequate care if the registry reported four DCM visits during which a PHQ-9 was administered.

### Data analysis

#### Randomized evaluation

We weighted all analyses to control for potential enrollment bias based on age and gender using administrative data on the approached population [34,44]. We weighted the analyses for attrition on baseline depression symptoms and functional status. We adjusted for possible clustering of data by site within region. Statistical analyses used STATA 10.0 [45] and SPSS 15.0 [46]. We also controlled for variation in elapsed time from baseline to follow-up surveys by including a variable indicating the number of days between the baseline and follow-up surveys.

We compared patient characteristics in CCM and non-CCM practices using t-tests for continuous variables and chi-square tests for categorical variables. For multivariate outcome analyses, we used generalized estimating equations (GEE) to assess the impact of CCM intervention at seven months after the baseline with repeated measures at the patient level, two records per person (pre- and post-intervention periods) [47]. The effect of the intervention was assessed by the interaction term of the indicator of post-time period and the indicator of the intervention group. We did not conduct three-level analyses that treated region as a blocking factor and examined CCM at the site level because we had only one usual care site per region. For continuous dependent variables (such as PHQ-9 score), we used the GEE model with the normal distribution and an identity link function. For dichotomous dependent variables (such as the indicator of adequate dosage of antidepressant use), we use the GEE model with a binomial distribution and a logit link function. For all the GEE models, we used the exchangeable correlation option to account for the correlation at the patient and clinic level. We compared CCM to non-CCM patient outcomes using two analytical models. In the first model, we included all covariates as individual variables. In the second model, we included only the DPI. Because the results were similar, we used the DPI model.

#### Care manager registry analysis

We used chi-square to assess the relationships between provider referral type and adequate care manager follow-up. We used one-way ANOVA to assess PHQ-9 difference scores with Scheffe post-hoc comparisons to show which level of follow-up by DCMs had the strongest relationships with PHQ-9 outcomes.

## Results

**Figure 1:**
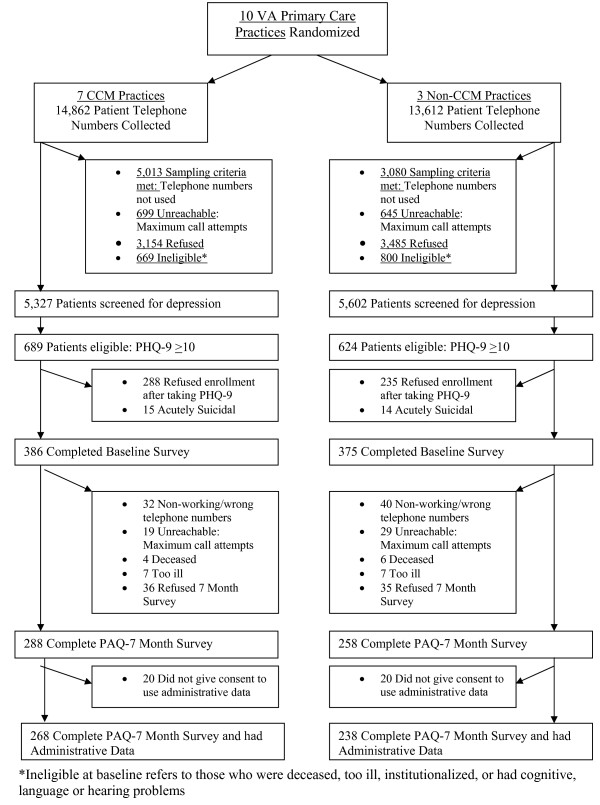
**Sampling flow chart**.

Figure 1 shows patient enrollment in the randomized evaluation. 10, 929 primary care patients were screened for depression, with 1, 313 patients scoring 10 or more on the PHQ-9. A total of 761 completed the baseline survey and, of those, 72% (546) completed the seven-month survey. Of those completing the follow-up survey, 93% (506) consented to have their VA administrative data used for research purposes.

Table 1 compares enrolled EBQI-CCM and non EBQI-CCM site patients at baseline and shows no significant differences. Completers of the seven-month survey were not significantly different from non-completers on any of these baseline patient characteristics.

**Table 1:** Self-reported characteristics at baseline of randomized evaluation-enrolled patients in EBQI-CCM versus non EBQI-CCM sites

Baseline patient characteristics	EBQI-CCM (N = 288)	Non EBQI-CCM (N = 258)	P-value
Age-mean (SD)	64.0 (12.4)	64.4 (12.7)	0.73
% Male (gender)	95.8	96.5	0.62
% white	86.2	88.1	0.53
% married	63.9	63.9	0.99
Education			0.36
% < high school	11.5	14.6	
% high school or more	88.5	85.4	
Employment			0.81
% Working	14.8	14.1	
% not working/on disability/retired/other	85.2	85.9	
Region			
% A	32.5	34.6	0.63
% B	36.2	34.7	0.72
% C	31.2	30.7	0.90
Seattle comorbidity index^41^	7.50 (3.3)	7.65 (3.4)	0.61
% 3 chronic conditions or more	16.6	12.7	0.22
% Current PTSD	50.9	49.1	0.62
Alcohol use (AUDIT_C)			
0	57.6	54.1	0.70
1 to 3	22.5	23.3	
4 to 12	19.9	22.5	
% ≥2 VA mental health visits (past 6 months)	27.0	26.3	0.85
% Poor health status	46.9	41.0	0.19
% Satisfied or very satisfied w/mental health care	62.4	67.2	0.27
Total social support - mean (SD)	2.27 (1.2)	2.32 (1.7)	0.64

Adjusted for population weights. N.S. = not significant at p < 0.05 level. Total social support - lower is more supportive

Table 2 shows the depression treatment and patient outcome results across all patients enrolled in the randomized evaluation at seven months. EBQI-CCM site patients were significantly more likely to have an adequate dosage of antidepressant prescribed than were non-EBQI-CCM patients (65.7% for EBQI-CCM versus 43.4% for non-EBQI CCM, p < 0.01). They were also significantly more likely to have filled an antidepressant prescription (MPR > 0). Completion of full appropriate care within the seven-month follow-up period, however, either through completion of appropriate antidepressants or psychotherapy, was not different between the groups. There was also no significant EBQI-CCM/non EBQI-CCM difference in terms of depression symptoms, functioning, or satisfaction with care. In exploratory multivariable regression results predicting seven-month PHQ-9 scores, EBQI-CCM also showed no significant effect on symptom outcomes. Significant predictors of seven-month PHQ-9 scores were the DPI prognostic index, baseline PHQ-9, and VA administrative region.

**Table 2:** Depression treatment and outcomes comparing EBQI-CCM site patients with non EBQI-CCM site patients at baseline and seven months

	Baseline			Seven months		
	EBQI-CCM	Non EBQI-CCM	Difference	EBQI-CCM	Non EBQI-CCM	Difference
**Clinical care (administrative data)**	**(N = 268)**	**(N = 238)**		**(N = 268)**	**(N = 238)**	
Adequate dosage of antidepressant prescribed within 7 months post baseline (%)	49.6	41.5	8.1*	65.7	43.4	22.3**
Medication possession ratio > 0 (%)	52	43	9	67	45	22*
Completion of appropriate care (MPR > 0.8 or completion of 4+ therapy visits) (%)	38.0	34.9	3.1	47.1	41.9	5.2
**Symptoms and functioning (survey data)**	**(N = 288)**	**(N = 258)**		**(N = 288)**	**(N = 258)**	
Depression symptom severity (mean PHQ-9 score)^†^(SD)	15.5 (4.4)	15.7 (4.7)	-0.2	11.5 (6.5)	11.6 (6.7)	-0.1
Patients below threshold for major depression (% PHQ-9 < 10)	0	0	0	39.9	41.4	-1.5
Physical functional status (mean SF-12 role physical score)^**†† **^(SD)	29.2 (36.2)	34.8 (40.7)	-5.6*	32.6 (39.4)	34.1 (35.6)	-1.5
Emotional functional status (mean SF-12 role emotional score)^**†† **^(SD)	47.1 (41.4)	50.0 (41.8)	-2.9	49.9 (49.3)	50.0 (41.5)	-0.1
Satisfaction with Mental Health Care (% somewhat or very satisfied)^**††**^	67.2	62.4	4.8	69.1	71.2	-2.1

Means, SDs and percentages are unadjusted. Analyses were weighted for enrollment bias and attrition.

* = p < 0.05

** = p < 0.01

**^† ^**Lower score is better

**^†† ^**Higher score is better.

### Effects of context: adherence to CMM protocols among randomized evaluation patients

Evaluation of adherence to CCM protocols showed delays in contacting and initiating treatment among patients referred by the study. Care managers initiated patient contact an average of 47 days after referral among randomized evaluation patients, and initiated treatment an average of 16 days after first contact (not shown).

**Figure 2:**
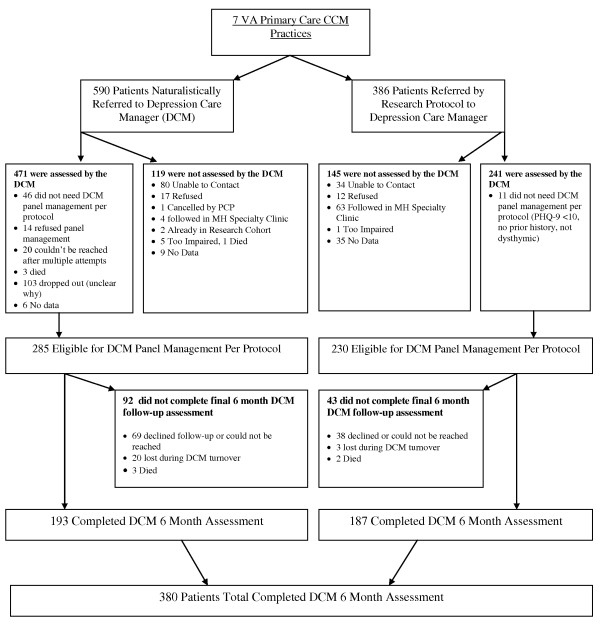
**Naturalistic and evaluation-enrolled collaborative care patient flow chart**.

Figure 2 shows that among the 386 randomized evaluation patients referred for care management, 241 (62%) had an initial clinical assessment by the DCM and 145 (38%) did not. Among the 241 patients assessed, 230 (95%) were eligible for care manager panel management per protocol, while 11(5%) were referred back to the primary care clinician with management suggestions only because they had PHQ-9s less than ten, no prior history of depression and no dysthymia. Among the 230 eligible for panel management, 187 (81%) completed the six month care manager assessment. Overall, considering the entire group of referred patients, 232 (60%) did not receive adequate care manager follow-up per the TIDES protocol. In addition to the 145 patients without an initial DCM clinical assessment, 87 (36%) of the 241 eligible patients did not receive adequate care manager follow-up (not shown).

### Effects of context: EBQI

All regions and target practices carried out priority setting followed by PDSA cycles and design and implementation of CCM. CCM as implemented included all aspects of the Chronic Illness Care model [48], including: redesign (hiring and training of a care manager); information technology (electronic consult and note tracking) [27-29]; education and decision support (care manager registries, standardized electronic assessment and follow-up notes, clinician pocket cards, educational sessions, academic detailing) [24,25]; collaboration with mental health specialty for education, emergencies, and care manager supervision [31]; identification of community and local resources; and patient self-management support. More detailed results can be found elsewhere [11,19]. Regions and practices varied, however, in the extent of leadership, staff, and clinician involvement [49].

### Effects of context: clinician adopter status

Table 3 shows the effects of clinician adopter status on patient completion of adequate care manager follow-up within the randomized evaluation. The patients in this table include the 241 randomized evaluation patients who had an initial care manager clinical assessment at baseline (Figure 2).

**Table 3:** Early adopter clinician effects on adequacy of care manager follow-up in EBQI-CCM sites

Patients' primary care clinicians' history of early adoption of collaborative care management (CCM)	EBQI-CCM site patients enrolled in the randomized evaluation and recorded in the care manager quality improvement registry*, ** (N = 241)		
	Patient received adequate care manager follow-up	Patient did not receive adequate care manager follow-up	Total
	N (%)	N (%)	N (%)
Evaluation-enrolled patients of 21 EBQI-CCM site clinicians with low predilection to adopt CCM (made no referrals) (34.4% of study clinicians)	35 (47.9)	38 (52.1)	73 (100)
Evaluation-enrolled patients of 17 EBQI-CCM slow CCM adopter clinicians (made 1 to 4 referrals) (27.9% of study clinicians)	36 (64.3)	20 (35.7)	56 (100)
Evaluation-enrolled patients of 11 early CCM adopter clinicians (made 5 to 9 referrals) (18.0% of study clinicians)	42 (77.8)	12 (22.2)	54 (100)
Evaluation-enrolled patients of 12 habitual user clinicians (made 10 or more referrals) (19.7% of study clinicians)	41 (70.7)	17 (29.3)	58 (100)
Total (evaluation-enrolled patients of all 61 clinicians)	154 (63.9)	87 (36.1)	241 (100)

*The quality improvement registry includes both a) patients enrolled in the randomized evaluation and referred to CCM by researchers and b) naturalistically referred patients. The registry records all visits for patients experiencing CCM. Only patients enrolled in the randomized evaluation who also are listed in the registry (and thus have data on CCM visits) are included in these analyses.

** p = 0.003 comparing adequate care manager follow-up by type of clinician, with the difference between clinicians with low predilection to adopt CCM and all others showing the greatest difference (Scheffe test)

Among the 241, 71% (41 of 58) of patients of CCM habitual users (making 10 or more referrals), 78% (42 of 54) of patients of CCM early adopters (making 5 to 9 referrals), 64% (36 of 56) of patients of CCM slow adopters, and 48% (35 of 73) of patients of clinicians with a low predilection to adopt CCM, received adequate care manager follow-up (p = 0.003). Results were similar if we conducted the analyses on the full population of 386 randomized evaluation patients referred to care management, and assigned those not reached by DCMs as not receiving adequate follow-up (p = 0.03 for the parallel comparison), or if we restricted the analyses to the 230 of 241 randomized evaluation patients who were eligible for panel management based on a baseline PHQ-9 by the DCM that showed probable major depression (p = 0.01).

### Effects of Context: Adherence to protocol among randomized evaluation versus naturalistically referred patients

Figure 2 suggests that EBQI-CCM was used differently under naturalistic provider-referred than under randomized evaluation-referred conditions, including differences in delays and rates of completion for baseline DCM assessment, types of patients referred and rates of completion of the DCM six month follow-up assessment. Among 976 total patients (randomized evaluation plus naturalistically referred) entered into the care manager registry preceding and during the evaluation enrollment period, 386 (40%) were referred through the randomized evaluation process and 590 (60%) were referred naturalistically. Among the 386 randomized evaluation-based referrals, 62% (241) completed a baseline assessment. Among the 590 naturalistic referrals, 80% (471) completed a baseline DCM assessment (p < 0.001 for differences in assessment). Compared to the average elapsed time of 47 days from referral to care managers' patient contact initiation for randomized evaluation patients, naturalistic referrals were contacted in an average of 15 days (p < 0.001 for differences in delays). Once assessed by the DCM, a greater proportion of randomized evaluation than of naturalistically referred patients were assessed as eligible for DCM panel management (230 of 241, or 95% of randomized evaluation patients compared to 285 of 471, or 61% of naturalistically referred patients (p < .0001 for differences in eligibility). Once enrolled in panel management randomized evaluation patients were more likely to complete the six month DCM follow-up assessment. Among the 230 randomized evaluation patients eligible for panel management, 187 (81%) completed a six month care manager assessment. Among the 285 naturalistically referred patients who were eligible for panel management, 193 (68%) completed a six month care manager assessment (p < .0005 for differences in six month assessments).

We found that CCM offered as a voluntary referral service to PCCs was heavily used by some clinicians and rarely used by others (not shown). Naturalistically referred patients came predominantly from early adopters and habitual users, while research-referred, randomized evaluation-enrolled patients reflected the full distribution of clinician adopter levels. For example, among the 386 randomized evaluation-enrolled patients referred for care management, 27.2% came from clinicians who had referred 10 or more patients to CCM (habitual users). Among 590 naturalistically referred patients, 72.5% came from clinicians who were habitual users of CCM (p < 0.001).

Finally, we assessed the relationship between depression symptom outcomes and adequate depression care manager follow-up. We found that 24-week PHQ-9 outcomes were significantly better among patients in EBQI-CCM practices (randomized evaluation referred and naturalistically referred patients combined) who received adequate care manager follow-up than among those who did not based on bivariate regression analysis with PHQ-9 change as the dependent variable (p = 0.001). As shown in Table 4, this result appears to reflect a dose response pattern for care manager visits, with the largest difference being between those with just baseline and 24-week follow-up visits and those with four or more visits (p < 0.001).

**Table 4:** Number of depression care manager visits versus change in patient PHQ-9 depression scores

Depression care manager visits during which a PHQ-9 was administered**	PHQ-9 score mean change (lower is better)	95% confidence intervals
(a) Baseline and 24 week only (2 total) (n = 95)	-3.83	-5.10, -2.56
(b) Baseline, 24 week, and one additional (3 total) (n = 120)	-6.94	-8.09, -5.79
(c) Baseline, 24 week, and two to four additional (4 to 6 total) (n = 163)	-8.45	-9.49, -7.41

Based on Care Manager Quality Improvement Registry Data. **Total n = 378 representing those clinician- and research-referred patients finishing panel management (380, Fig. 2) minus two clinician-referred patients who completed the 24 week DCM follow-up visit but did not have a 24 week DCM PHQ-9 recorded**. Significance levels are p < 0.001 comparing the PHQ-9 change for group (a) to group (c); p = 0.003 comparing group (a) to group (b); and p = 0.16 comparing group (b) to group (c).

## Discussion

This study aimed to determine whether healthcare organizations could improve depression care quality using EBQI for adapting CCM to local and organizational context [8-11]. This multi-level research/clinical partnership approach called for systematic adaptation of previously-tested, effective CCM for depression [4-7], and for focusing on the key aspects of the Chronic Illness Care Model (*i.e*., patient self-management support, decision support, delivery system design, and clinical information systems) [48]. Researchers served as technical experts rather than decision makers or implementers. To rigorously and formatively evaluate EBQI-CCM early in its life cycle in VA, we used cluster randomized trial methodology powered to detect changes in the process, but not outcomes, of care. Our answer regarding whether healthcare organizations can use EBQI methods to improve depression care is mixed in that EBQI-CCM showed improvements in process (antidepressant use) while not demonstrating outcome improvements.

Unlike our answer regarding the effectiveness of EBQI-CCM, our answer regarding the usefulness of randomized trials for formative evaluation is strongly positive. The trial was relatively inexpensive ($750, 000) relative to system costs for fully implementing CCM nationally; identified the need to improve the EBQI-CCM model; and provided critical information on how to improve it. Information on issues raised by this trial, such as care manager workload, follow-up, access, and clinician effects on patient outcomes, is essential if the results of over 35 CCM randomized trials are to be replicated in routine care.

To understand study results, we evaluated adherence to the study intervention plans. In this study, the investigators implemented EBQI, rather than CCM itself; engaged sites implemented CCM. In terms of overall adherence to EBQI methods, our approach engaged regional and local leaders effectively in guiding local QI teams through PDSA cycles for designing and implementing CCM. All participating practices implemented and maintained EBQI-CCM throughout the study. In terms of adherence to CCM, as implemented by regional and local leaders with EBQI support, QI data [11] showed that EBQI-CCM incorporated the key features identified in the published CCM literature [50] in terms of patient education and activation [26,51], care management follow-up, systematically assessed symptoms, and collaboration between primary care providers, care managers, and mental health specialists [31,32]. Adherence to the TIDES DCM protocol for promptness and completion of all required clinical assessments for individual randomized evaluation patients, however, was problematic.

Despite the identified problems with completion of assessments, the EBQI-CCM site patients were prescribed antidepressants at appropriate doses significantly more often than those in non EBQI-CCM practices (a 23% difference). EBQI-CCM site patients similarly had significantly more prescriptions actually filled (a 22% difference). Prior studies of CQI or lower-intensity EBQI for depression in primary care have not shown improved prescribing [8,14,15]. Increased antidepressant use, however, did not translate into robust improvements in depression symptoms, functional status, or satisfaction with care in intent to treat analyses.

Because we aimed for the most efficient use of study resources for evaluating the process and effectiveness of an implementation method, rather than the effectiveness of CCM, we predicated our design on the lower sample size required for assessing a key process of care (antidepressant use) rather than the more demanding sample size needed for assessing effects on patient symptom outcomes. Our power to detect a 20% difference in depression symptoms was only between 0.21 and 0.29, based on the obtained sample size and ICC. We thus cannot definitively say that depression symptom outcomes did not improve within the timeframe studied. We were, however, disappointed in the lack of robust symptom impacts, and sought to determine more explicitly what lessons readers should take away from our work.

To place our findings in context, we first asked: Do our randomized evaluation results test the effectiveness of CCM as a model? We conclude they do not. Other CCM studies have tested the CCM model as implemented using designs with higher researcher control, and shown effectiveness in diverse healthcare sites [4-7]. These studies, however, did not test self-implementation of CCM by healthcare practices or sites using QI methods. For example, meta-analyses on the effectiveness of the CCM model [4-6] exclude prior studies of QI methods for implementing CCM [8,14,15], recognizing that these studies address a different question. Our randomized evaluation results test the ability of healthcare organizations and sites to adapt and implement research-designed CCM as a part of their organizational cultures and structures. In so doing, the results provide information for improvement. Because typical healthcare organizations or practices must use QI methods to adopt research-based depression care models, the challenges faced by this study are likely to be relevant to managers, policymakers, and researchers interested in improving depression care at a system or organizational level.

Our goal of combing a randomized evaluation with QI methods resulted in challenges related to timing. Our fixed windows for baseline and follow-up surveys meant that delays in initiation of care management for study patients, followed by lags in PCC ordering of treatments, were not accommodated in assessing outcomes. Thus, for many patients, antidepressant treatment or psychotherapy began only a short time before the seven-month follow-up survey. Furthermore, while 81% of randomized evaluation patients eligible for panel management completed the six month DCM assessment, many fewer had completed the designated number of follow-up contacts by that time. These results highlight the challenges for researchers in timing outcome measures relative to patient access to CCM in a study with a rigorous randomized design but low researcher control of the intervention.

Excessive demand for care management proved to be another challenge, and one that is potentially relevant to CCM program managers. While we did not intend to overload care managers, we inadvertently did. As originally envisioned, the randomized evaluation would have begun after EBQI-CCM practices had completed a small number of PDSA cycles of the CCM intervention involving as few as ten and no more than fifty total patients. Under this scenario, care managers could have covered both naturalistic referrals and randomized evaluation referrals, given typical care manger caseloads [52]. In reality, the requirements of eight separate IRBs, faced with an unfamiliar implementation research model and with the introduction of HIPAA [53], led to a prolonged period between start of naturalistic PDSA intervention development and start of the randomized evaluation (a gap per intervention practice of between 111 and 334 days with a mean of 263 days). The study team discovered that it was not feasible, under QI conditions, to turn off naturalistic referrals. Thus, care manager caseloads were full with naturalistically referred patients prior to the start of the randomized evaluation.

Our study mimicked a potential organizational policy such that patients screening positive for depression would be automatically referred to CCM, along with patients referred by their PCCs. This scenario represents a potentially realistic policy in VA in particular, because routine depression screening is already mandated. Our results show that implementing effective follow-up of primary care-detected depression for all eligible patients will be challenging.

Few previous studies of CCM have tested reach [54] or the degree to which all eligible individuals in a given clinical setting can have access to the CCM model. Most previous CCM randomized trials have limited total patient access to CCM to patients enrolled in the trial, thus artificially controlling demand. Care manager caseload capacity [52] bounds effective reach under naturalistic conditions. Our study ratio of approximately one care manager per 10, 000 primary care patients may need to be adjusted or ameliorated by other depression care redesigns.

Looking within the randomized evaluation and its patients, we found unanticipated associations between patient outcomes and whether the PCC they belonged to was an early adopter of CCM. Patients referred to CCM by the study team, but whose PCC was an early CCM adopter [20], were significantly more likely to be assessed by care managers and to receive adequate care manager follow-up than other patients, independent of patient depression severity or comorbidities. These results suggest that patient access to full CCM care was shaped more by who their provider was than by patient need.

We think the clinician effects we observed are likely to substantially affect CCM models under naturalistic conditions. Clinician effects did not influence patient enrollment in the study. No clinicians in these practices refused to have their patients referred to CCM by study personnel, and the proportions of randomized evaluation patients belonging to early adopter clinicians versus those with a low predilection to adopt the model mirrored the proportions of clinicians who fell into these categories in the study practices.

Clinician predisposition to adopt CCM affects use of the model under naturalistic conditions in another way as well. In essence, patients of early adopter clinicians tend to monopolize the DCM resource. For example, 73% of naturalistically referred patients belonged to clinicians who habitually used CCM (ten or more naturalistic referrals), while only 27% of randomized evaluation patients belonged to this type of clinician. Registry data on patients naturalistically referred to CCM shows the same pattern we saw in data from the randomized evaluation; the quality of CCM care is better among patients of early adopter clinicians [11]. Differential use of and benefits from CCM resources based on clinician characteristics should be taken into account both in interpreting studies of CCM and in implementing CCM as routine care.

Theories of social justice and mental health parity would argue that patient need, rather than clinician characteristics, should govern access to clinical resources. Our results emphasize the importance of active PCC engagement in CCM, a topic not addressed in most prior CCM research, though identified as issue by prior qualitative work on the TIDES program [49]. Clinician effects may be mediated by, for example, more effective encouragement to patients about participating, and greater promptness in responding to care manager suggestions about ordering treatment. These activities might in turn be moderated by differences in clinician knowledge and attitudes about depression and/or greater experience in using CCM. Monitoring of clinician effects on use of CCM (and potentially other mental health services) is critical for CCM programs, and better methods for bringing all clinicians in line with the early adopters are needed [55].

There are reasons to collect program evaluation data other than testing program efficacy or effectiveness. We think CCM programs should monitor patient outcomes using a registry, as was done in this study, for purposes of monitoring program utility and safety. Unlike our randomized evaluation results, our pre-post care manager registry data reflects how the program functions naturalistically. These naturalistic data, while not testing model effectiveness, show that CCM as implemented in these VA sites performed safely, and with outcomes that met or exceeded CCM targets for patients whose clinicians chose to refer them.

We used our registry data as well as our randomized trial data to shape and improve EBQI-CCM. As reported elsewhere [11,19], registry results on naturalistically referred patients show that: 82% of 208 were treated for depression in primary care without specialty referral; 74% stayed on medication for the recommended time; and 90% of primary care managed patients and 50% of mental health specialty managed patients had clinically significant reductions in depressive symptomatology (PHQ-9 scores < 10) at six months. On average, PHQ-9 scores improved nine points; improvement remained significant controlling for depression severity and complexity. While subject to selection bias, these results showed potential benefit of the program for diverse patients. If the results had been different, such as showing little or no symptom improvement or raising safety concerns, we would have considered stopping or fully redesigning the program. Instead, our follow-up PDSA cycles focused on reducing patient and clinician selection effects while continuing to monitor patient outcomes.

Our comparison of registry data with rigorous study data is encouraging regarding the validity of registry data for depression program monitoring purposes. Although we would not have discovered the effects of clinician adopter status based on registry data, because of the few included patients belonging to low adopter clinicians, we can replicate this clinician effect in retrospect through the registry. Other major context effects demonstrated by the randomized experiment, including effects of patient complexity, can also be observed through the registry data, supporting its validity. Per protocol analysis (looking only at patients who received full CCM) of symptom and functional status outcomes for randomized evaluation patients was consistent with registry results in terms of the level of outcome improvement observed, providing qualitative triangulation on the importance of ensuring patient completion of CCM as a critical target for improvement. We conclude that registry data on patient care and symptom outcomes can be accurate enough for program monitoring, with appropriate attention to selection bias and care manager training on data collection.

Registry data has limitations in addition to its inability to provide fully representative data. We know that registry patients were selected and not representative of all eligible patients. In addition, care managers, though extensively trained, may have been less objective or consistent in their administration of the PHQ-9 than were external data collectors.

One of the explicit goals of the TIDES program was to develop a CCM model that could be spread nationally in VA [56]. In terms of this goal, the randomized evaluation of EBQI reported here informed the ongoing QI and model spread process for TIDES. For example, the issues with differential PCC involvement led to systematic training and engagement approaches on a national basis [56]. As it improved, the TIDES program became one of the bases for the Veterans Health Administration (VHA) Primary Care-Mental Health Integration(PC-MHI) initiative [57-59] and is codified in the VHA's Uniform Mental Health Services Package directive [60]. EBQI thus seems to be an effective method for designing a program that sustains and spreads. Data from the randomized evaluation reported here, however, indicate that ensuring that the sustained, spread programs produced by EBQI achieve comparative effectiveness on a population basis is also critical. Ongoing national evaluation of primary care-mental health integration in VA has the potential to achieve this goal.

This study has limitations. The study focused on a single healthcare system (the VA), and on non-academic, small to medium-sized practices, almost a third of which were rural [21]. Results may not be generalizable to other systems or practice types. Second, our study's power to detect symptom outcomes was limited by our follow-up window of seven months. For some patients, delays in access to care managers and thus in treatment initiation may have limited the possibilities for completing treatment within the study window. Third, we were not able to confirm registry data on care manager visits by analysis of administrative data because a specific encounter code for depression care management was not introduced by the VA until after this study. Fourth, use of consecutive sampling over-represents more frequent users of primary care relative to the full population of visiting patients [61]. Finally, our process evaluation subgroup analyses on early adopters are not appropriate for drawing conclusions about causality or the overall effectiveness of EBQI-CCM. Selection bias, in particular, cannot be eliminated as a factor in these analyses.

In summary, this study showed that CCM, as implemented using EBQI, improved antidepressant prescribing across a representative sample of patients attending study practices. While this randomized evaluation does not test the effectiveness of CCM as an ideal model, it does test the effectiveness of CCM as designed and implemented in VA using QI methods, albeit early in the program's lifespan. The study encountered a number of difficulties likely to apply to other healthcare organizations implementing CCM as routine care, including the consequences of care manager overload and of differential PCC adoption of CCM. The lack of robust patient symptom improvement for the experimental group compared to usual care points to the importance of continuously monitoring and improving CCM programs during and after implementation. Otherwise, the cost-effectiveness benefits promised by CCM studies will not be achieved in reality.

## Competing interests

The authors declare that they have no competing interests.

## Authors' contributions

EC, LR, CL, EY, BS, and BF substantially contributed to the conception and design. CB, ML, AL, and JU contributed to the analysis and interpretation of the data. All authors were involved in drafting the manuscript and/or critically revising it. All authors read and approved the final manuscript.
